# A Rejoinder to "A Friendly Cross-Examination."

**Published:** 1920-05-29

**Authors:** Philip A. Inman

**Affiliations:** Secretary of the Hospital Saturday Fund


### THE HOSPITAL SATURDAY FUND.

### A Rejoinder to "A Friendly Cross-examination."

By Philip A. Inman, Secretary of the Hospital Saturday Fund.

To be catechised is not always a pleasant experience. But surely the most ardent or super-sensitive of its supporters can find no occasion to be perturbed by the cross-examination of the Hospital Saturday Fund which appeared in The Hospital of the 22nd instant. Constructive criticisms and helpful questions should always be welcomed, especially so when they relate to the work of a public organisation, and it is obvious, from a perusal of the article under review, that the writer has approached his subject, not with a desire to discourage or embarrass, but rather to render assistance to the Hospital Saturday Fund in its work for the voluntary hospitals of the Metropolis. It is a pleasure, therefore, to acknowledge this service and to respond to his desire for further information.

Before proceeding to deal seriatim with the points raised by your correspondent, may I refer to the only paragraph in my letter of the 4th instant, which produces no comment? This relates to the Fund's progress during recent years. In the year 1914 the income was £40,676, in 1919 it reached £74,848, an increase of 87½ per cent. This result is the more remarkable when it is remembered that during these years thousands of honorary workers were serving in His Majesty's Forces, and innumerable appeals were issued on behalf of many patriotic funds. The suggestion might be made that the changing currency accounted for the increase. The records of the Fund prove that this is not so. The rate of subscription in 1919 was identically the same as in 1914. The more immediate progress of the Fund is even more satisfactory. During the first three months of the current year the amount received exceeds that of the corresponding period of last year by £3,000. This represents an increase of 60,000 subscribers. "Nothing succeeds like success," and it is confidently anticipated that the Fund will extend until its ambition to have a collection in every place of business in the City is reached.

References are made by the special correspondent to the support given direct by employés of firms to their local institutions. He admits that a precise allowance would have to be made for these collections in any estimate of the Fund's activities, and he proceeds to ask: "What is this large number?" I hope shortly to be in possession of this information. Already a survey has been made, and the result is interesting and instructive. The analysis of one road, which is typical of many others, is as follows:

| | No. |
|---|---|
| Workshops, factories, and business houses ... ... | 31 |
| " where collections are taken for the Hospital Saturday Fund ... ... ... | 14 |
| " where collections are taken for one of the local hospitals ... ... ... ... ... ... | 9 |
| " where alternative collections are taken for the Hospital Saturday Fund and the local hospitals ... ... ... ... ... ... | 5 |
| " where, so far as can be ascertained, no hospital collections are made ... ... | 3 |

During the year 1918 twelve London hospitals, whose balance-sheets were studied at random, state that they received from workmen's collections £7,319. These figures, whilst not complete, afford some indication of the contributions made direct to the hospitals by the workers, and I think your correspondent will agree that my statement is justified, "The employés of a large number of firms contribute direct to their local hospital."

Your correspondent discovers from the figures supplied that the "average number of subscribers to each collection is twenty." The amount realised from a collection of this character would be approximately £3 10s. per year. One of the smallest collections is that of a workshop in the City where there are three employés. In twenty years the Fund has received from this particular source £13. Is a collection of this kind worth the trouble? There is only one answer. Many a mickle makes a muckle. "It is not by huge donations, but by the thousands of trifles from willing hearts that the burden of the hospitals will be lightened." The Hospital Saturday Fund acknowledges with gratitude the efforts of the twos and threes.

In accordance with the desire expressed, I send a map showing the grouping of the thirty-three Hospital Saturday Fund local committees. It will be observed that these committees are founded in most of the Metropolitan boroughs, and their influence and activities extend far and wide.

Your correspondent continues, "Is a subscription of 3s. 9d. a year satisfactory?" It is when two facts are remembered: (1) It is well known that the Hospital Saturday Fund works on the principle of the 1d. per week collection. A year's subscription therefore realises 4s. 4d.; but (2) The employés of some firms, as already pointed out, contribute alternatively, one week to the Hospital Saturday Fund, and the next to their local institution. Consequently your correspondent is incorrect when he states "the direct subscriptions to a local hospital do not affect this result."

Happily, the sole supporters of the Chelsea Football Club are not "the sole or chief group of footballers provided with an opportunity to contribute." A perusal of the annual report reveals the fact that considerable financial help is received by the Fund from many similar sources. There are, in addition, several charity cup competitions, the entire proceeds of which are devoted to the Fund.

Lastly, it is agreed that the question of most interest at the moment is the attitude of the Fund towards the future. I referred in my previous communication to the many opportunities there are for advancement. The Fund is under no delusion concerning the work yet to be accomplished. The summit is not reached, but the steep hill which was commenced in 1873 will be mounted. The Hospital Saturday Fund programme is not "mark-time," but "Excelsior," and my Council is happy in the knowledge that its aims and objects have only to be known to be supported. If the outcome of the publicity increases the knowledge it will be a matter for gratitude.

